# The Treatment of Varicose Ulcers

**Published:** 1891-06

**Authors:** 


### THE TREATMENT OF VARICOSE ULCERS.

Dr. J. Braun states that a large experience with this class of cases has convinced him of the superior advantages of a 10 per cent. ointment of zinc in lanoline (zinc oxid. 15.0, lanoline 110.0, ung. emoll. 40.0). This is applied as follows in cases of ulcer of the legs: The surface of the ulcer is thoroughly washed with lukewarm water, carefully dried with a compress, and the salve spread on a soft piece of linen applied to the sore and retained by a handkerchief or strip of linen. The patient remains in bed until the sores have cicatrized The good effects of this treatment soon become apparent, the pains and itching disappear, and the profuse watery secretion is arrested, the greater part of the transuded fluid being absorbed by the lanolin which has hygroscopic properties. The ointment forms a protective covering under which healing takes place, while the lanolin by virtue of its antiseptic powers prevents decomposition of the secretions. In cases of unhealthy ulcers, the application should be renewed four or five times daily for the first few days. After three or four days, the surface of the sore will be found much cleaner, and cicatrization will have occurred at the margins, and then the ointment need only be applied three times daily. Once a day, and preferably in the morning, the ulcer should be irrigated with lukewarm water and dried, and before each application any remaining secretion is removed with absorbent cotton. Since employing this treatment the author has not found it necessary to resort to skin transplantation.—*Allg, Wein. Medizin. Central-Zeitg.*, Feb. 3, 1891.—*International Jour. Sarg.*, April, 1891.

PRURITIS ANI.—Ohmann Dumeniel (*St. Louis Clinique*), April, 1891:

Gives his plan of treatment as follows : Correct constipation or diarrhoea; build up especially the nervous system with such as phosphorous, strychnia, arsenic, etc. He alternates with the following formulas :

℞ Syr. hypophosphite, Co. (Fellows) ℥ jv.

Sig. A teaspoonful in water four times a day.

After a time, the following is ordered :

℞ Liquor kali arsenitis, . . ʒ ijss.

Vini ferri, . . . . . ℥ jv.

M. Sig. A teaspoonful in water after meals.

When this has been used a sufficient length of time administer

℞ Strychniæ sulphat., . . gr. j.

Ferri ædacti.

Quinæ bisulphat., . . . aa ʒj.

M. ft. massa et divide in pil. No. 60. Sig. One pill three times a day.

In addition are general reconstructives and anodynes to make sleep, if needed.

*Local Treatment.*—Treat ulcers, hemorrhoids or diseases of rectum, as well as fissures, excoriations, hemorrhoids or growths around anus. If much thickening of skin around anus, apply pure creasote locally. Pain lasts but a short time and is followed by relief. Follow by applying, night and morning, an antipruritic antiparasitic lotion. He often uses with success:

℞ Hydrargyr. bichlorid. . . gr. jss.

Ammon. muriat. . . . . gr. i j.

Acid carbolic, . . . . ʒ j.

Glycerini,. . . . . . ℥ i j.

Aquæ rosæ q. s. ad. . . . ℥ vj.

M. Sig. Apply locally.

The carbolic acid may be varied to suit case.

Chlorophenique often renders good service, being antiparasitic, antipruritic as well as slightly anodyne.

